# Crop improvement using life cycle datasets acquired under field conditions

**DOI:** 10.3389/fpls.2015.00740

**Published:** 2015-09-22

**Authors:** Keiichi Mochida, Daisuke Saisho, Takashi Hirayama

**Affiliations:** ^1^Cellulose Production Research Team, Biomass Engineering Research Division, RIKEN Center for Sustainable Resource Science, Yokohama, Japan; ^2^Gene Discovery Research Group, RIKEN Center for Sustainable Resource Science, Yokohama, Japan; ^3^Kihara Institute for Biological Research, Yokohama City University, Yokohama, Japan; ^4^Group of Genome Diversity, Institute of Plant Science and Resources, Okayama University, Kurashiki, Japan; ^5^Group of Environmental Response Systems, Institute of Plant Science and Resources, Okayama University, Kurashiki, Japan

**Keywords:** population genomics, transcriptome, epigenome, crop phenology, machine learning

## Abstract

Crops are exposed to various environmental stresses in the field throughout their life cycle. Modern plant science has provided remarkable insights into the molecular networks of plant stress responses in laboratory conditions, but the responses of different crops to environmental stresses in the field need to be elucidated. Recent advances in omics analytical techniques and information technology have enabled us to integrate data from a spectrum of physiological metrics of field crops. The interdisciplinary efforts of plant science and data science enable us to explore factors that affect crop productivity and identify stress tolerance-related genes and alleles. Here, we describe recent advances in technologies that are key components for data driven crop design, such as population genomics, chronological omics analyses, and computer-aided molecular network prediction. Integration of the outcomes from these technologies will accelerate our understanding of crop phenology under practical field situations and identify key characteristics to represent crop stress status. These elements would help us to genetically engineer “designed crops” to prevent yield shortfalls because of environmental fluctuations due to future climate change.

## Introduction

Abiotic stress conditions can have a negative effect on the productivity of agricultural systems. According to a recent report from the Intergovernmental Panel on Climate Change (IPCC), humanity is facing an increased risk of agricultural production shortfalls (https://www.ipcc.ch/report/ar5/). Modern plant science has achieved remarkable advances in elucidating the molecular systems associated with abiotic stress responses in plants under artificially controlled conditions inside the laboratory. This is especially true for the model plant species *Arabidopsis thaliana*, where functional genomic analyses after the completion of sequencing its genome have identified key genes involved in the regulatory network of abiotic stress responses (Hirayama and Shinozaki, 2010; Nakashima et al., 2014). However, several critical problems remain regarding the practical application of this laboratory-derived knowledge to molecular science based breeding of crops adapted to adverse environments. The next challenge in generating practical stress-tolerant crops that can withstand future climate changes requires an understanding of the responses of crops to multiple abiotic stresses under field growth conditions. Large fluctuations in multiple abiotic stress conditions and large heterogeneity between stress levels for different plant genotypes and developmental stages are the chief causes of the complexity underlying variations in abiotic stress responses in crops under field conditions (Mittler and Blumwald, 2010).

The considerable recent advances in analytical technologies in omics-based research have provided crucial resources for investigating biological systems not only in model plants but also crop species (Mochida and Shinozaki, 2010). With large-scale transcriptome datasets, it will be feasible to perform correlated gene expression analyses to identify candidate genes involved in particular gene networks (Mochida et al., 2011; Obayashi et al., 2014). Metabolome analyses provide information on the accumulation patterns of metabolites in plants in various biological contexts, such as changes in environment, developmental stage, and genotype, and offer an efficient approach to revealing the metabolic systems underlying complex phenotypes (Tohge et al., 2011; Balmer et al., 2013; Fukushima and Kusano, 2013). Hormonomic analysis, which enables simultaneous profiling of phytohormones and their derivatives, also plays an important role in investigating phytohormone networks in different biological contexts (Kojima et al., 2009; Kanno et al., 2010). Integrated approaches using synergistic combinations of different omics systems, so called “*trans*-omics,” are increasingly an effective means of investigating plant cellular systems in response to abiotic stresses (Dinakar and Bartels, 2013; Deshmukh et al., 2014). Furthermore, in the last decade, rapid progress in next-generation sequencing (NGS) technologies has enabled access to genome-scale sequence information from a wide range of organisms, even those with large and complex genome structures such as wheat and barley (Mochida and Shinozaki, 2011, 2013). Whole genome resequencing is a feasible NGS application for exploring genome-scale polymorphisms in natural variations, and to identify the association between genetic polymorphisms and phenotypic variations including those induced by stress. Another NGS application, RNA-seq, is highly scalable and can be used to rapidly acquire comprehensive transcriptome data in any species. The effective use of genome-scale datasets from various types of omics analyses rely on computer-aided approaches that have become increasingly important in studies to determine the responses of plant cellular systems to environmental changes. A broad range of bioinformatics techniques are essential to access large-scale omics datasets and to efficiently discover biologically significant information and then use this to answer specific questions on stress responses in plants. Systems approaches with mathematical modeling have recently received much attention for understanding biological phenomena under both controlled laboratory conditions and fluctuating field conditions.

**FIGURE 1:**
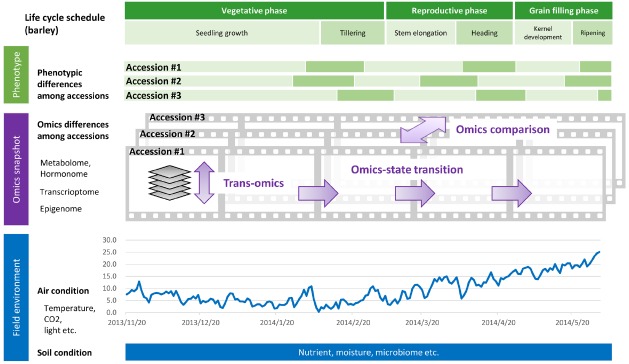
**Phenology datasets during the life cycle of a crop grown under field conditions.** Time series omics data such as transcriptome, epigenome, and metabolome/hormonome data can be acquired as sequential “snapshots” throughout the crop life cycle. Parameters regarding field environment including air and soil conditions can be acquired as “streamed” datasets.

With the currently available methods and resources for studying plant stress responses, it is expected that interdisciplinary efforts involving plant science and data science will enable exploration of factors that affect crop productivity and will aid discovery of genes and alleles associated with quantitative traits of stress tolerance in crops. It is essential not only to examine a snapshot of the cellular network under multiple stress conditions at a particular moment but also to monitor throughout the life cycle, since changes in physiological status over time might influence the eventual phenotype. The identification and estimation of the effects of parameters, based on an understanding of the genetics and physiology of responses to environmental changes of crops throughout their life cycle, are required to design a crop with the required performance of stress tolerances in the field condition. In this mini review, we provide an overview of recent advances in technologies that are key components for data driven crop design, such as crop population genomics, chronological *trans*-omics analysis, and computer-aided molecular network prediction (Figure 1).

## Population Genomics in Crops

Genetic diversity in a crop population is a valuable resource for identifying alleles that can be exploited to improve crop productivity under a variety of adverse conditions (Huang and Han, 2014). Population-wide molecular phylogeographic analysis of a crop species can provide molecular evidence on its demographic history as a domesticated species (Saisho and Purugganan, 2007). Additionally, such analysis may identify relationships between biased geographic distributions and genetic differentiation, such as the particular genotype associated with a trait providing adaptation to a particular local environment. For example, in barley, a population-wide analysis of bio-geography and the degree of vernalization requirement showed a biased geographic distribution pattern of a quantitative growth habit trait (Saisho et al., 2011). As another example, a population-scale evolutionary analysis of *HvAACT1*, which encodes a citrate transporter involved in aluminum tolerance in barley and has a 1-kb insertion for Al-tolerance in the upstream region, only occurs in Al-tolerant cultivars in Japan, Korea, and China, suggesting adaptation to the acid soils of these areas (Fujii et al., 2012). These examples in barley demonstrate that population-scale exploration of the association between geographic distributions and genotypes could be an efficient strategy to identify alleles for locally adapted traits. The development of NGS has allowed high-throughput genotyping such as whole-genome re-sequencing, genotyping by sequencing (GBS), RNA-seq based genotyping, and exome sequencing, to rapidly generate genome-scale datasets on genetic polymorphism.

Whole genome re-sequencing analysis with information from a reference genome is a straightforward method to characterize genome-wide polymorphism patterns among accessions. Representative accessions, for example, elite lines in tomato, soybean, maize, and rice, have been investigated by whole genome re-sequencing, which has identified useful resources for further genetic studies in each crop (Lai et al., 2010; Arai-Kichise et al., 2011; Subbaiyan et al., 2012; Causse et al., 2013; Li et al., 2013). In some species with smaller genomes, the whole genome re-sequencing approach has been applied to population-wide analyses of genome-wide polymorphism patterns; this approach has been employed in poplar tree, tomato, common bean, and rice (Evans et al., 2014; Lin et al., 2014; Schmutz et al., 2014). Huang et al. (2012) carried out a whole-genome resequencing analysis in wild rice populations to generate a genome variation map, which also provided insights into the domestication history of domesticated rice. More recently, a core collection of 3000 rice accessions from 89 countries were re-sequenced and 18.9 million single nucleotide polymorphisms (SNPs) were found (Li et al., 2014a). A whole-genome resequencing dataset on a population wide scale can provide an important resource especially for understanding the demographic history of a domesticated species, and facilitate recognition of alleles associated with adaptive phenotypic variations, for example, tolerance of particular environments, by applying the resequencing dataset together with a dataset of the trait based on a genome-wide association study (GWAS).

Genotyping by sequencing or RNA-seq based genotyping are more affordable approaches than whole genome sequencing for genome-wide and population wide genotyping. GBS is a popular method that provides a rapid and robust approach for identifying sequences with a low level of representation in multiplex samples (Elshire et al., 2011; Poland et al., 2012). A number of genome-wide polymorphism datasets have been obtained from GBS analysis, for example, 2815 accessions of the USA national maize inbred seed bank using 681,257 SNPs (Romay et al., 2013), 971 worldwide accessions of sorghum with ∼265,000 SNPs (Morris et al., 2013), and 304 short-season soybean lines with >47,000 SNPs (Sonah et al., 2015); these have also been applied to GWAS analysis (so called GBS-GWAS analysis).

High-throughput genome-scale genotyping is a key technology to finding adaptive genes that might be of promise for improving crop productivity in particular environments. Careful analysis of associations between genome-wide patterns of polymorphism and phenotypic variations in adaptive traits holds great promise for elucidating crop species domestication histories at both the ecological and evolutionary levels (Huang and Han, 2014). Furthermore, such analyses enable the estimation of the genetic effects of candidate allelic combinations and quantification of heritability, which are critical parameters to production of reliable allelic combinations in the designed crop varieties.

## Omics-Based Elucidation of Crop Phenology

Crops in the field are exposed to multiple environmental stimuli. Crop life cycle changes are often triggered by environmental signals, for example, temperature- and photoperiod-related cues for flowering, and timely initiation of these developmental changes is critical to final productivity. Therefore, understanding the physiological responses of crops to seasonal and short-term fluctuations in the environment is vital to estimation of their potential impact on the crop life cycle and eventual yield. For this purpose, omics-based long-term chronological profiling of crops under field conditions is an efficient strategy for characterizing phenological responses in gene regulatory networks. Such analyses provide insights into the regulation of gene functions in response to environmental fluctuations and are an aid for the identification of genes that are key mediators between environmental signals and crop productivity (Gibson, 2008).

Time-series transcriptome analysis during plant life cycles has become an efficient approach to infer phenological responses under variable environmental conditions. Richards et al. (2012) performed a time-series transcriptome analysis in *A. thaliana* shoots in the field from seedling to reproductive stages and found enrichment of several co-expressed gene clusters that were induced by abiotic and biotic stresses. Several studies have investigated the dynamics of genome-scale gene expression patterns using transcriptome analysis of a life cycle sample series from cultivated rice plants grown under field conditions (Sato et al., 2011; Nagano et al., 2012; Matsuzaki et al., 2015). It was shown that mathematical modeling and prediction of genome-wide transcriptional changes under field conditions could be successfully carried out based on life cycle transcriptome datasets and meteorological datasets (Nagano et al., 2012). Similarly, the transcriptome in a single clone of a grapevine cultivar was recorded over three consecutive years in 11 vineyards and it was demonstrated that the additive effects of temperature and water availability particularly influenced grape quality (Dal Santo et al., 2013).

Gene expression in response to developmental and environmental signals are often regulated by epigenetic mechanisms through small RNAs, histone modifications and DNA methylation (Chinnusamy and Zhu, 2009; Kinoshita and Seki, 2014). Recent studies in plants have shown that epigenetic mechanisms are involved in some important biological processes such as genomic imprinting, defense responses to pathogens, acclimation to abiotic stresses, and vernalization responses (Ikeda, 2012; Kim et al., 2012; Woods et al., 2014; Liu et al., 2015). Furthermore, some of these epigenetic modifications are inherited through mitotic and meiotic cell divisions. The meiotically heritable epigenetic modifications are termed “epialleles” and can cause heritable phenotypic variation (Kalisz and Purugganan, 2004; Weigel and Colot, 2012). In epigenetic regulation of plant stress tolerance, non-heritable epigenetic modifications are involved in acclimation as a short-term stress resistance response. Mitotically and meiotically heritable epigenetic modifications function as a “stress memory” within and across generations, respectively (Chinnusamy and Zhu, 2009). A recent study of epigenetic recombinant inbred lines (epiRILs) of *A. thaliana* showed that variations in DNA methylation cause heritable variation of ecologically important plant traits, such as root allocation, drought tolerance and nutrient plasticity (Zhang et al., 2013). Plant epigenome data are therefore vital to the understanding of epigenetic and genetic regulation of phenotypic diversity (Schmitz et al., 2013). It is now recognized that epigenetic diversity in populations and epigenetic changes in response to environmental fluctuation are also considerable factors in adaptation and evolution and could be a resource for improvement of crop stress tolerance.

Phenome analyses provide datasets on a variety of phenotypes using mutants and/or natural variants. With large-scale loss-of-function or gain-of-function mutants, phenome analyses using artificially induced mutants have played an essential role in discovering genes involved in phenotypic changes and for determining their biological functions (Kuromori et al., 2006, 2009). Recent advances in technologies such as sensors, imaging, and internet communication have begun to provide various tools for high-throughput plant phenotyping under field conditions (Klukas et al., 2014; Li et al., 2014b; Fahlgren et al., 2015; Grosskinsky et al., 2015). Remote phenotyping of crops in the field is emerging as a feasible application for drones with multiple sensors, not only for trait analysis in genetics but also for precision agriculture (Liebisch et al., 2015). Hand-held devices that aid phenotyping can be an efficient tool to carry out high-throughput phenotypic data acquisition (Vankudavath et al., 2012). Integration of imaging and sensing technologies have provided tools for non-invasive approaches to monitor biometrics of growing crops (Busemeyer et al., 2013; Li et al., 2014b; Kjaer and Ottosen, 2015). High-throughput plant phenotyping approaches have been synergistically applied to genetics to accelerate gene discovery in crops. For example in rice, a high-throughput rice phenotyping facility (HRPF) makes it possible to monitor 15 traits during the rice growth period; and these data can be applied to GWAS (Yang et al., 2014). In addition to conventional phenotyping, quantitative molecular profiles from various high-throughput analytical techniques such as metabolomics could be used as a comprehensive dataset of molecular phenotypes.

Metabolome analysis provides a comprehensive molecular snapshot based on metabolites synthesized in biological reactions. It can be affected by various factors, such as genetic and epigenetic factors, developmental stages and organs, environmental stimuli and diseases. Therefore, it could be thought that the metabolome can represent chemical phenotypes reflecting the physiological state in an organism (Mochida et al., 2009; Sakurai et al., 2013). The combinatorial use of high-throughput metabolome profiling and GWAS has become an efficient strategy to reveal the genetic architecture of biochemical properties in plants (Adamski and Suhre, 2013; Wen et al., 2014; Matsuda et al., 2015). Metabolome profiling at different plant developmental stages can provide stage-dependent information on the physiological state of the plant in response to the environment during the lifecycle (Onda et al., 2015). Therefore, chronological metabolome analysis throughout the plant life cycle under field conditions will also be a vital strategy to describe the physiological state and to extract state factors associated with traits in crops.

## Computer Aided Understanding of Biological Phenomena in Plants

As described above, recent advances in omics analytical technologies have produced a wealth of genome-scale datasets even from crops growing in field conditions. One of the important issues in bioinformatics is how to deal with such large and heterogeneous datasets, and to establish heuristic procedures to accelerate gene discovery (Mochida and Shinozaki, 2011). Information resources such as databases and computational tools are extremely important for effectively handling genome-scale datasets. Additionally, data storage for omics datasets must ensure persistence and retrieval functionalities for shared use (Mochida and Shinozaki, 2011).

To gain a mechanistic understanding of biological systems, mathematical modeling and simulation approaches have been applied to the study of plant cellular metabolism, growth, developmental processes, and responses to the environment (Aikawa et al., 2010; De Vos et al., 2012; Katsuragi et al., 2013; Satake et al., 2013; Miyazaki et al., 2014). Mathematical modeling approaches are also used to understand a wide range of biological functions in growth, survival, and reproduction in plants, for example, in the circadian regulation of plant carbohydrate metabolism (Webb and Satake, 2015), phloem sucrose transport associated with rice grain yield (Seki et al., 2015), and silicon uptake in rice roots (Sakurai et al., 2015).

**FIGURE 2:**
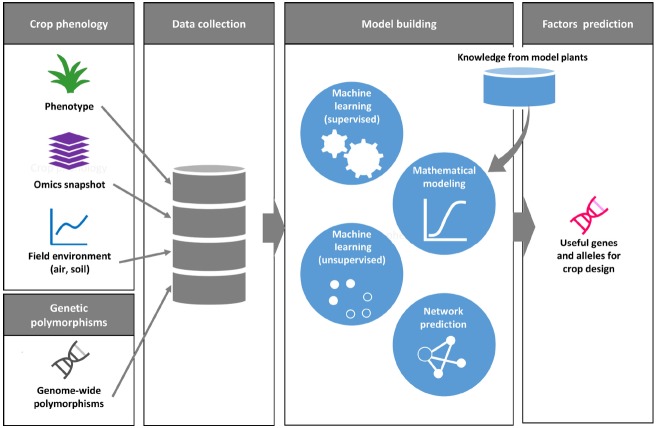
**A conceptual framework for phenology data driven crop design.** Phenology datasets include plant phenotypes, omics snapshots with environmental data. A genome-wide polymorphism dataset is useful to find genetic association between accessions with traits as well as with physiological state. For model building, the collected data are applied to various types of computer-aided methods. Digitized datasets can also be assessed against data on well-characterized gene functions in model plants. Machine learning-based data clustering and network prediction may help us to identify candidate genes and alleles for crop improvement.

Machine learning is a field of computer science for the design of computational algorithms that automatically improve with experience. In the last two decades, this research field has dramatically advanced with the emergence of artificial intelligence and data science, and has been applied in various fields in science, technology and commerce (Jordan and Mitchell, 2015). Machine learning is also used in applications for the analysis of genome-scale datasets and other large-scale omics datasets in life science (Libbrecht and Noble, 2015). Learning methods in machine learning are usually classified into two primary categories of supervised and unsupervised learning. The supervised machine learning aims to produce an algorithm to predict output on unknown input via a training process using a dataset of known pairs of input and output. The unsupervised machine learning methods are used to extract structures and identify their features in a given dataset without examples for training. Computational modeling using machine learning has been performed recently with the aim of predicting gene networks based on large-scale transcriptome datasets in plants. For example, in *Arabidopsis*, supervised machine learning was used to build a network model of responses to stress conditions, to explore genes related to stress responses, and to predict molecular interactions (Ma et al., 2014; Nourani et al., 2015). Machine learning provides a data-driven approach to extract latent rules or patterns from a comprehensively collected dataset without any biased view on the biological phenomena of interest (Figure 2).

## Conclusion and Future Perspectives

Cross-disciplinary research, including computer science, functional genomics, and crop phenology, will provide a unique opportunity to establish technologies for data-driven crop design to prevent crop yield shortfall under changing future environments. It is expected that a synergy of life science and data science will allow us to perceive novel and latent values underlying the observed dataset by unbiased data-driven analyses. Unbiased illustration of physiological state dynamics of crops growing under field conditions could be an efficient strategy to figure out features of genetic factors but also “state factors” that determine eventual agronomical traits. Another issue for the data-driven approach is how we fill the gap between findings from model plants studied in laboratories and those from crops under field conditions to generalize our knowledge on plant systems including those in response to environmental changes. Complementary use of hypothesis-driven and data-driven approaches should be a practical way for further understanding of physiological responses to field environments with cross-referencing to knowledge from model plants that has been accumulated in laboratories. Therefore, platforms for computing and linking life science data will also play more significant roles in research on data-driven crop breeding.

## Author Contributions

KM, DS, and TH conceived research and wrote the manuscript.

### Conflict of Interest Statement

The authors declare that the research was conducted in the absence of any commercial or financial relationships that could be construed as a potential conflict of interest.
